# Development of the Korean Medicine Core Outcome Set for Stroke Sequelae: Herbal Medicine Treatment of Elderly Patients With Stroke Sequelae in Primary Clinics

**DOI:** 10.3389/fphar.2022.868662

**Published:** 2022-04-25

**Authors:** Jiyun Cha, Sungha Kim, Pyung-Wha Kim, Hesol Lee, Mi Mi Ko, Soobin Jang, Myeong Soo Lee

**Affiliations:** ^1^ Digital Health Research Division, Korea Institute of Oriental Medicine, Daejeon, South Korea; ^2^ Department of Internal Korean Medicine, College of Korean Medicine, Daejeon University, Daejeon, South Korea; ^3^ KM Science Research Division, Korea Institute of Oriental Medicine, Daejeon, South Korea; ^4^ R&D Strategy Division, Korea Institute of Oriental Medicine, Daejeon, South Korea; ^5^ Department of Preventive Medicine, College of Korean Medicine, Daegu Haany University, Gyeongsan, South Korea

**Keywords:** core outcome set, stroke sequelae, elderly, primary care, herbal medicine, Korean medicine

## Abstract

**Objectives:** We developed a Korean medicine core outcome set for stroke sequelae (COS-SS-KM) to evaluate the effectiveness and safety of herbal medicine (HM) for stroke sequelae, especially for elderly stroke patients in primary clinics.

**Methods:** We identified previously reported outcomes from a literature review and defined the list of outcomes and effect modifiers for the core outcome set (COS) questionnaire. Three rounds of modified Delphi consensus exercises with experts were conducted online for suitability assessment, and one round of a modified Delphi consensus exercise with primary clinicians was conducted for feasibility assessment.

**Results:** The review identified 17 outcomes and 16 effect modifiers; moreover, six outcomes and one effect modifier were suggested by the experts. The final COS comprised 8 outcomes and 12 effect modifiers for history taking, and experts listed 13 major symptoms of stroke sequelae for symptom assessment. The clinicians agreed on the feasibility of the COS.

**Conclusion:** This COS will help primary care researchers assess the effectiveness of pharmacotherapy, including HM, for elderly patients with stroke sequelae. Future studies should focus on reflecting the opinions of all stakeholders.

## 1 Introduction

Stroke-induced neurological injury leads to multiple sequelae throughout life. These post-stroke symptoms known as a result of the tissue injury are not easily resolved by time (Becker, 2016; De Doncker et al., 2018). A systematic review (SR) showed that 74% of patients with stroke have at least one unmet need after hospital discharge, including cognitive problems, emotional/mood problems, fatigue, and pain (Chen et al., 2019). Comprehensive sequelae of stroke lead patients to experience decreased quality of life and increased risk of additional hospitalization (Naess and Nyland, 2021).

Herbal medicine (HM) could have potential benefits on post-stroke symptoms, including aphasia and depression (Zeng et al., 2017, Zhang et al., 2018). A network meta-analysis showed that HM was effective for recovery after stroke (Han et al., 2017). A recent review reported that HM natural compounds including glycosides, flavonoids, phenols, and terpenes exhibited neuroprotective/neurorestorative effect against stroke (Zhu et al., 2022). For instance, Astragaloside IV from *Astragalus membranaceus* has been revealed to reduce neuronal apoptosis and infarct volume, leading to decreased neurological deficits (Zhang et al., 2019; Yin et al., 2020). Activation of neurogenesis-associated signaling pathways and down-regulating inflammation and apoptosis pathways are revealed as key mechanisms of HM in stroke sequelae treatment (Shen et al., 2015).

In Korea, HM based on Korean Medicine (KM) is the preferred primary care treatment for patients with stroke sequelae. However, studies on its therapeutic effect are still lacking. Despite their value in reflecting the realities of community care settings, clinical trials in primary or KM clinics have limitations (Lindbloom et al., 2004). Lack of manpower and equipment limits researchers in primary or KM clinics from conducting studies with the same research design as those in academic hospitals. This leads to low-quality studies investigating inadequate outcomes, thereby reducing the reliability of the studies’ conclusions.

In 2020, the Korean government launched a pilot project that aimed to support herbal medicinal treatment for patients (aged >65 years) with stroke sequelae in primary clinics. Healthcare insurance supports the cost of 10 days of HMs that meet the government’s standards of diagnosis and quality (No, 2020). However, large-scale pilot studies or standard outcome models for KM treatment have not yet been proposed.

This study aimed to develop a Korean medicine core outcome set for stroke sequelae (COS-SS-KM), to evaluate the safety and effectiveness of herbal KM treatments for stroke sequelae in primary clinics. A core outcome set (COS) refers to an agreed-upon, standardized set of outcomes recommended by the Core Outcome Measures for Effectiveness Trials (COMET) initiative. Adopting a suitable COS enhances the quality of clinical research (Williamson et al., 2012). A literature review and modified Delphi exercises with stakeholders were conducted to develop the COS.

## 2 Methods

We developed a COS with relevant scope and rationale for the aforementioned purpose (Table 1). In addition, effect modifiers were extracted to provide items for patient history-taking guidelines. The COS development process was guided by the COMET initiative and reported based on the COS standard guidelines (Cadogan et al., 2015; Kirkham et al., 2016; Rankin et al., 2018). COS development was conducted in three phases: Phase 1 (generation and refinement of a comprehensive list of outcomes and effect modifiers from a literature review), Phase 2 (modified Delphi consensus exercise by experts), and Phase 3 (modified Delphi consensus exercise by primary clinicians).

**TABLE 1:** Recommendations of the Core Outcome Set Standards for Development (COS-STAD).

Domains	No	Methodology	Notes
Scope specification	1	The research or practice setting(s) in which the COS is to be applied	The COS will be applied in research studies for primary KM clinics
	2	The health condition(s) covered by the COS.	The disease covered by the COS is sequelae of stroke
	3	The population(s) covered by the COS.	The target patients are adults aged >65 years with stroke sequelae
	4	The intervention(s) covered by the COS.	The intervention covered by the COS is HM of KM.
Stakeholders involved	5	Those who will use the COS in research	KM researchers in primary clinics will use the COS for clinical trials
	6	Healthcare professionals with experience of patients with the condition	Experts of KM stroke therapy and KM clinicians in primary clinics participated to the COS development
	7	Patients with the condition or their representatives	Patients of stroke sequelae did not participate
Consensus process	8	The initial list of outcomes considered both healthcare professionals’ and patients’ views	The initial list of outcomes included in the COS is identified through a literature review
	9	A scoring process and consensus definition were described a priori	A Delphi survey and consensus meeting was adopted to select the outcomes
	10	Criteria for including/excluding/adding outcomes were described a priori	(1) Delphi with experts: 4-point Likert scale and review of all panels, inclusion criteria was the unanimity
			(2) Delphi with primary clinicians: 9-point Likert scale and calculation of CVR.
	11	Care was taken to avoid ambiguity of language used in the list of outcomes	The language and medical terms in our COS ensure uniformity of the outcome terms

COS, core outcome set; KM, Korean medicine; HM, herbal medicine; CVR, content validity ratio.

### 2.1 Phase 1: Generating and Refining a Comprehensive List of Outcomes and Effect Modifiers

In this phase, a project management group (PMG) was selected, and a comprehensive list of outcomes and effect modifiers were identified for the Delphi exercise in Phase 2. The PMG was composed of five researchers of the Korea Institute of Oriental Medicine (JC: literature review, outcome/effect-modifier extraction, removal of duplicated results; SK: literature review, outcome/effect-modifier extraction; PWK, HL, and SJ: result review). Prior to the literature review, the PMG clarified the scope of the COS.

First, a literature review was conducted to identify published SRs and randomized controlled trials (RCTs). One researcher (JC) conducted search on an English database [Medline (*via* PubMed)] and a Korean database (the Oriental Medicine Advanced Searching Integrated System). The search was on studies published from 2016 to 2020 and there was no restriction on language, publication country, or status. The following terms were used when searching PubMed: 1) (Traditional Chinese Medicine OR herbal medicine) AND geriatrics AND [systematicreview (Filter)], 2) [Traditional Chinese Medicine (MeSH) OR herbal medicine (MeSH)] AND geriatrics AND [systematicreview (Filter)] (Supplementary Material S1).

From the searched list, a researcher included SRs that contained trials of HM for geriatrics with stroke. Two researchers (JC and SK) independently assessed whether the searched articles met the inclusion criteria and hand-searched reference lists of the included SRs to identify the original RCTs and measured outcomes. They extracted the inclusion criteria, target symptoms, and outcomes from the included studies. In case of disagreement, they resolved it through discussions.

Second, two researchers examined the recommended outcomes and effect modifiers for stroke sequelae by reviewing books related to stroke and neurologic rehabilitation, such as “Stroke” (Korea Stroke Society, 2015), “Cardiovascular and Neurological Medicine in Korean Medicine II” (AKMPCNM, 2018), and “Stroke Rehabilitation” (Gillen, 2016). Third, two researchers examined outcomes for KM primary clinics by reviewing related articles and reports (Park, 2003; Lee, 2017). One researcher screened the extracted outcomes and removed duplicated studies and outcomes. Subsequently, the PMG reviewed the list and removed outcomes outside the scope of this COS to produce the final list.

### 2.2 Phase 2: Modified Delphi Consensus Exercise With Experts

The outcomes and effect modifiers derived from Phase 1 were condensed through three rounds of modified Delphi consensus exercises by the expert panel.

#### 2.2.1 Recruitment of the Delphi Panel

Potential participants were sought through official contact by the PMG with the Society of Stroke on Korean Medicine, which recommended members for inclusion in the panel. Inclusion criteria for potential expert participants were as follows: being a professor of KM at a university, or being a clinician in a KM hospital specializing in the care of elderly patients with stroke.

Seven potential participants that met the criteria were recommended. The PMG introduced the aim and scope of the COS *via* email and invited them to partake in the modified Delphi consensus exercise. All of them replied to the email and participated in the Delphi consensus.

#### 2.2.2 The Modified Delphi Questionnaire

This study included questionnaires (distributed from November to December 2020) with group feedback and meetings for consensus. The group feedback was embedded in subsequent questionnaires to inform respondents about the views of all participants. Each questionnaire along with the study’s background information was distributed *via* email. Participants had 1 week to complete each round.

In Round 1, participants received a list of outcomes and effect modifiers identified in Phase 1 and were asked to rate the importance of each. To avoid the tendency of choosing the middle, the PMG modified the Grading of Recommendations Assessment, Development and Evaluation working group scale recommended by the COMET guidelines to a 4-point numerical scale. Scores of 1, 2, 3, and 4 represented outcomes or effect modifiers of “rarely important,” “sometimes important,” “important but not critical,” and “critical,” respectively. During Round 1, participants were allowed to suggest additional outcomes or effect modifiers not presented in the list as well as ideas for optimizing the COS for KM primary care. Regarding the analysis of Round 1, the score distribution was calculated (i.e., the percentage of participants that rated each outcome and effect modifier at each score). Suggested outcomes or effect modifiers were reviewed by the PMG members to remove those beyond the scope of the COS.

Round 2 and Round 3 were conducted through videoconferencing. In Round 2, the panel defined the characteristics of the optimal outcomes for KM primary care and selected the final outcome candidates and effect modifiers. Round 2 questionnaires comprised all outcomes and effect modifiers included in Round 1, as well as suggested outcomes, effect modifiers, and ideas put forth by the panel in Round 1. Moreover, each outcome and effect modifier was re-discussed while considering the group’s responses and their previous responses. Final COS candidates included only those on which all participants had consensually agreed.

In Round 3, the participants reviewed the results of the previous round and discussed the outcomes or effect modifiers without reaching a consensus. Subsequently, they reviewed all outcomes and effect modifiers to determine those suitable for the scope of the COS. Discussion continued until a consensus was reached regarding the COS.

### 2.3 Phase 3: Modified Delphi Consensus Exercise With Primary Clinicians

The feasibility of derived outcomes and effect modifiers were reviewed through one round of modified Delphi consensus exercise by the primary clinicians, one of the key stakeholders.

#### 2.3.1 Recruitment of the Delphi Panel

Potential participants were sought through official contact by the PMG with the Korean Medicine Specialist Association, and panelists were recruited among their members. All recommended participants were KM primary clinicians with at least 5 years of clinical experience.

Potential participants were initially contacted *via* an email that introduced the aim and scope of the COS and invited them to participate in the modified Delphi consensus exercise. All potential participants replied to the email. Eleven clinicians were selected to participate, of which six specialized in stroke and geriatric diseases.

#### 2.3.2 Modified Delphi Questionnaire

We asked the clinicians to review the derived outcomes and modifiers and assess their feasibility in terms of collection in KM primary clinics. The questionnaire comprised five sections, as follows: 1) effect modifiers for general health, 2) effect modifiers in terms of stroke history, 3) modified Measure Yourself Medical Outcome Profile 2 (MYMOP2), 4) patient satisfaction, 5) and the five-level EuroQol 5-D (EQ-5D-5L), which includes the EuroQol Visual Analogue Scale (EQ-VAS). The clinicians responded to each question on a 9-point scale.

### 2.4 Data Analysis

#### 2.4.1 Phase 2: Modified Delphi Consensus Exercise With Experts

The criteria for consensus in the COS were decided *a priori* as unanimity in Round 3. Consensus regarding the inclusion of an outcome in the COS (“consensus in”) was reached after Round 2 or Round 3 when a unanimous decision was reached. All other scores were classified as “no consensus;” such outcomes were considered ineligible for inclusion in the COS (Rankin et al., 2018).

#### 2.4.2 Phase 3: Modified Delphi Consensus Exercise With Primary Clinicians

As a primary outcome, the content validity ratio (CVR) proposed by Lawshe (1975) was used to measure content validity and consensus formation. The CVR was calculated as follows:

$$CVR=\frac{N_{e}-\left(\frac{N}{2}\right)}{\frac{N}{2}}$$

where *N*_*e*_ is the number of experts who select 6–9 points, and *N* is the total number of experts.

A consensus was considered to have been reached if the CVR was >0.5, and the CVR critical value (the minimum CVR value at which the CVR can be considered to not to be consensual by chance) was considered to exclude accidental results and to determine how many panel members must agree to decide whether an item is essential. According to an existing study, the CVR critical value suitable for 11 Delphi panelists—the number in this study—was ≥0.636 (Ayre and Scally, 2014).

As secondary outcomes, the degree of consensus and degree of convergence were used to indicate the degree to which a consensus was reached, supplementing the CVR.

The degree of consensus was calculated as follows:

$$consensus=1-\;\frac{Q_{3}-Q_{1}\;}{Median},Q1:1st\;quartile,Q3:3rd\;quartile$$

The degree of convergence was calculated as follows:

$$consensus=\frac{Q_{3}-Q_{1}\;}{2}$$

In our study, when the degree of consensus was ≥0.75 and the degree of convergence was ≤0.5, agreement among panelists was considered to be achieved (Jang and Kwon, 2014).

### 2.5 Ethics and Consent

The Institutional Review Board of the Korea Institute of Oriental Medicine, Daejeon, Republic of Korea, provided an exemption for ethical approval. The participants provided their written informed consent to participate in this study.

## 3 Results

### 3.1 Phase 1: Generating and Refining a Comprehensive List of Outcomes and Effect Modifiers

During the literature review, 56 studies were searched from English and Korean databases. Three duplicated studies and 52 studies outside the inclusion criteria were excluded. The final selected one SR (Takayama and Iwasaki, 2017) included 5 RCTs (Supplementary Figure S1 and Supplementary Table S1). Furthermore, this SR (Takayama and Iwasaki, 2017) referred to the information about previous published SR (Siddiqui et al., 2013) which included 4 eligible RCTs (Supplementary Figure S1 and Supplementary Table S1). Therefore, a total of 9 RCTs was analyzed as listed in Supplementary Table S1.

The outcomes and detailed trial information were extracted from the 9 RCTs. In the PMG review, eight RCTs in which an inclusion criterion was stroke onset within 6 months were excluded, and outcomes from those trials were also excluded. Two outcomes (constipation scoring system and gas volume score) irrelevant to stroke were excluded. In total, five outcomes were included during the PMG review. During the review of books (Korea Stroke Society, 2015; Gillen, 2016; AKMPCNM, 2018), 11 outcomes and 16 effect modifiers related to stroke sequelae were extracted. All of these were included during the PMG review. From the articles on the development of appropriate outcomes for KM primary (Park, 2003; Lee, 2017), one outcome was extracted. This outcome was included during the PMG review.

In Phase 1, as a result, 17 outcomes and 16 effect modifiers were included in the consensus exercise (Phase 2). During the PMG review, the wording of these outcomes and effect modifiers were modified, as appropriate.

### 3.2 Phase 2: Modified Delphi Consensus Exercise With Experts

Seven experts in the fields of stroke and KM with ≥10 years of clinical experience were included in the Delphi panel. All participants responded to Round 1 (100%) and suggested a rationale for the COS (Table 1); moreover, the participants suggested six outcomes and one effect modifier.

**FIGURE 1:**
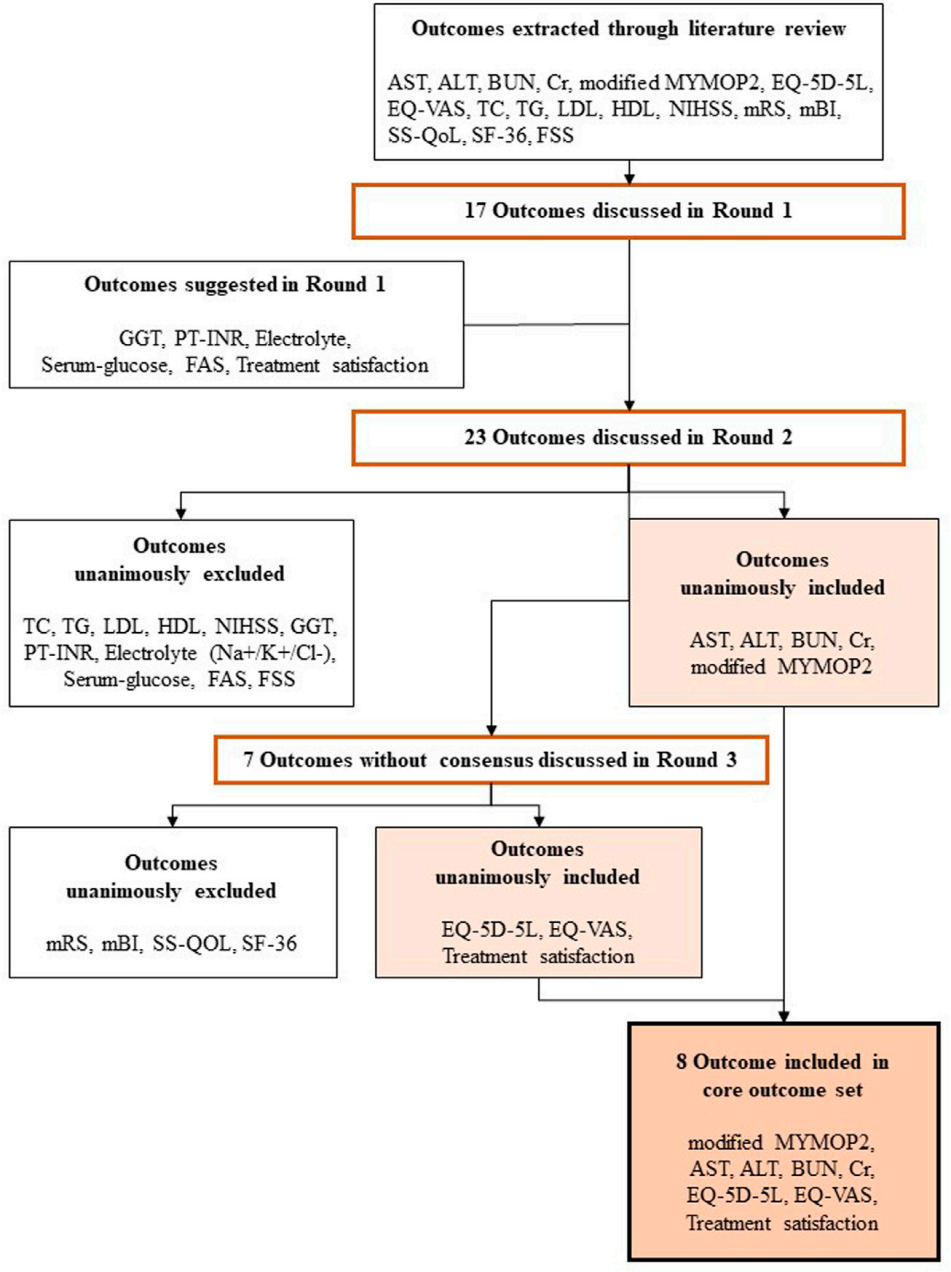
Overview of the development of the Korean Medicine Core Outcome Set for Stroke Sequelae for (COS-SS-KM). The expert panels reached a consensus regarding the core outcome set through three rounds. In Round 2 and Round 3, the inclusion criteria for each outcome were unanimous. AST, Aspartate Transminase; ALT; Alanine Transaminase; BUN, Blood Urea Nitrogen; Cr, Creatinine; MYMOP2, Measure Yourself Medical Outcome Profile 2; TC, Total Cholesterol; TG, Triglyceride; LDL, Low Density Lipoprotein; HDL, High Density Lipoprotein; NIHSS, National Institute of Health Stroke Scale; mRS, modified Rankin Scale; mBI, modified Barthel Index; SS-QoL, Stroke Specific Quality of Life; EQ-5D-5L, five-level EuroQol 5-D; EQ-VAS, EuroQol Visual Analogue Scale; SF-36, 36-item Short Form health-survey questionnaire; FSS, Fatigue Severity Scale; GGT, Gamma-glutamyl transferase; PT-INR, Prothrombin Time International Normalized Ratio; FAS, Fatigue Assessment Scale.

Each additional outcome was discussed by the PMG for consideration for inclusion in Round 2. The Round 2 questionnaire comprised all additional outcomes and modifiers, along with all outcomes and modifiers included in Round 1. All respondents to Round 1 were invited to participate in Round 2, of whom four participated. In Round 2, five outcomes and six effect modifiers that were unanimously agreed on for inclusion were determined to be “consensus in,” and there was agreement that the items with no consensus would be revisited in Round 3. Accordingly, round 3 questionnaires comprised outcomes and effect modifiers without a reached consensus. As in Round 2, all respondents to Round 1 were invited to participate in Round 3, of whom four participated. Three outcomes and six effect modifiers were determined to be “consensus in.” In addition, experts suggested offering a supplementary list of major stroke sequelae symptoms to help patients answer the modified MYMOP2. A total of 18 symptoms were suggested, of which 13 were agreed upon for inclusion. Finally, eight outcomes and 12 effect modifiers met the consensus inclusion criteria for the COS. Table 2 and Figure 1 present the process involved for each round.

**TABLE 2:** Summary of the consensus process during the three Delphi questionnaires.

Category	Item	Result	Note
Outcomes	AST	In	
	ALT	In	
	BUN	In	
	Cr	In	
	Modified MYMOP2	In	
	EQ-5D-5L	In	
	EQ-VAS	In	
	Treatment satisfaction	In	
	TC	Out	Difficult to assess in KM primary clinic
	TG	Out	Difficult to assess in KM primary clinic
	LDL	Out	Difficult to assess in KM primary clinic
	HDL	Out	Difficult to assess in KM primary clinic
	NIHSS	Out	Difficult to change with 10-day intervention
	mRS	Out	Difficult to change with 10-day intervention
	mBI	Out	Difficult to change with 10-day intervention
	SS-QoL	Out	Burdensome due to the large number of questions
	SF-36	Out	Burdensome due to the large number of questions
	FSS	Out	Less directly related to target disease
	GGT	Out	Difficult to assess in KM primary clinic
	PT-INR	Out	Difficult to assess in KM primary clinic
	Electrolyte (Na+/K+/Cl-)	Out	Difficult to assess in KM primary clinic
	Serum-glucose	Out	Less relevant except diabetes patients
	FAS	Out	Less directly related to target disease
Effect modifiers for history taking	Weight	In	
	Height	In	
	Onset	In	
	Type of stroke	In	
	Associated disease	In	
	Maintenance of rehabilitation treatment	In	
	Family history of stroke	In	
	Nutrition	In	
	Concomitant drug	In	
	Emergency history in acute stage	In	
	Neurologic severity in acute stage	In	
	History of drinking/smoking	In	
	Vital signs	Out	Less correlated with evaluation of KM treatment effect
	HbA1C	Out	Less relevant except in diabetes patients
	Lesion of stroke	Out	Difficult to assess in KM primary clinic
	Radiologic data	Out	Difficult to assess in KM primary clinic
	Socioeconomic status	Out	Less correlated with the evaluation of KM treatment effect
Symptoms for modified MYMOP2	Dysesthesia and central pain	In	
	Dyspepsia and constipation	In	
	Insomnia	In	
	Depression	In	
	Headache	In	
	Dizziness	In	
	Patient’s choice (free answer form)	In	
	Cognitive disorder	In	
	Dysphagia	In	
	Dysuria	In	
	Delirium	In	
	Dyspnea	In	
	Hemiplegia	In	
	Ataxia	Out	Difficult to change with 10-day intervention
	Other pains	Out	Less directly related to target disease
	Activities of daily living	Out	Difficult to change with 10-day intervention
	Fatigue	Out	Less directly related to target disease
	Language disorders (Dysarthria, dysphasia)	Out	Difficult to change with 10-day intervention

AST, aspartate transaminase; ALT, alanine transaminase; BUN, blood urea nitrogen; Cr, creatinine; AE, adverse event; MYMOP2, measure yourself medical outcome profile 2; EQ-5D-5L, five-level EuroQol 5-D; EQ-VAS, EuroQol visual analogue scale; FIM, functional independence measure; FMA, Fugl-Meyer assessment scores; DTER, diagnostic therapeutic effects of apoplexy; MCA, middle cerebral artery; ECG, electrocardiogram; TC, total cholesterol; TG, triglyceride; LDL, low density lipoprotein; HDL, high density lipoprotein; NIHSS, national institute of health stroke scale; mRS, modified Rankin scale; mBI, modified Barthel index; SS-QoL, stroke-specific quality of life; SF-36, 36-item Short Form health-survey questionnaire; FSS, fatigue severity scale; GGT, gamma-glutamyltransferase; PT-INR, prothrombin time-international normalized ratio, FAS, fatigue assessment scale.

### 3.3 Phase 3: Modified Delphi Consensus Exercise With Primary Clinicians

As the CVR of all questions exceeded the CVR critical value, a consensus was reached that all items were appropriate for inclusion in the COS and feasible for collection in KM primary clinics. The feasibility of collecting effect modifiers concerning stroke history and patient satisfaction met the criteria for the degree of consensus and convergence, indicating little deviation of opinion concerning collection of the effect modifiers. The feasibility of the modified MYMOP2, EQ-5D-5L, and EQ-VAS showed an insufficient degree of consensus and convergence, indicating relative controversy for collection (Table 3).

**TABLE 3:** Results of the modified Delphi consensus exercise with primary clinicians.

Question	Mean	Median	CVR	Degree of consensu	Degree of convergence
Effect modifiers about general health	5.45	6.00	0.82	0.75	0.75
Effect modifiers about stroke history	6.18	6.00	1.00	0.83	0.50
Modified MYMOP2	6.00	6.00	0.64	0.67	1.00
Patient satisfaction	7.73	8.00	1.00	0.88	0.50
EQ-5D-5L and EQ-VAS	5.36	6.00	0.64	0.58	1.25

CVR, critical value of ≥0.636—suitable for 11 panelists—was judged to suggest consensus among the panelists. A degree of consensus of ≥0.75 and a degree of convergence of ≤0.5 were judged that agreement among panel experts is achieved.

CVR, content validity ratio; MYMOP2, measure yourself medical outcome profile 2; EQ-5D-5L, five-level EuroQol 5-D; EQ-VAS, EuroQol visual analogue scale.

## 4 Discussion

### 4.1 Summary of the Main Results

**FIGURE 2:**
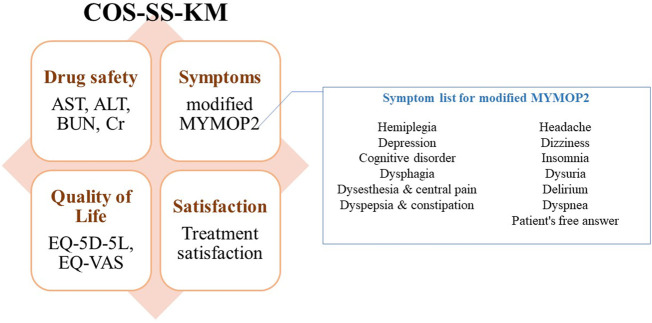
Conclusion of for the Korean Medicine Core Outcome Set for Stroke Sequelae (COS-SS-KM) development. Eight outcomes categorized in symptoms, drug safety, quality of life, and satisfaction were included in COS-SS-KM. For modified MYMOP2, symptom list suggested by experts will be used for response convenience. AST, aspartate transminase; ALT, alanine transaminase; BUN, blood urea nitrogen; Cr, creatinine; MYMOP2, Measure Yourself Medical Outcome Profile 2; EQ-5D-5L, EuroQol-5-Dimensions-5-Level; EQ-VAS, EuroQol Visual Analogue Scale.

The aim of this study was to develop a COS of HM for elderly patients with stroke in primary clinics. According to the guidelines of the COMET initiative, a literature review by the PMG and a modified Delphi exercise by clinical experts in KM treatment for stroke were conducted. Delphi exercises by KM primary care clinicians were conducted to review the feasibility of the COS. As a result, effect modifiers were obtained for patient history taking. A consensus-based list of outcomes and supplementary symptoms were obtained. The final outcome set for COS-SS-KM is shown in Figure 2.

### 4.2 Considerations for Core Outcome Set Development

With regards to outcome selection, we considered the characteristics of the elderly, chronic disease, and primary care.

Studies on the elderly should consider their unique physical and mental characteristics. Elderly individuals, especially those with physical or cognitive impairments, present with poor comprehension and increased fear for outcome assessment. Therefore, outcomes for the elderly should have a wide measurement scale, be simple and quick, and involve non-invasive measurement procedures that do not require much cooperation. Moreover, for complicated physical conditions of the elderly, measures for functional performance and quality of life (QOL) should be considered (Faes et al., 2007).

Although chronic diseases are slow and are not treated as an emergency, they affect the patient’s QOL by requiring long-term treatment. Traditional oriental medicinal treatment, including KM, seeks to improve both pathological symptoms and general health, including vitality, and is effective for chronic diseases. Therefore, as a post-KM-treatment outcome for chronic patients, it is recommended that QOL, overall health-related symptoms, and disease-specific symptomatic changes are assessed (Megari, 2013).

Approximately 83% of KM institutions are primary care clinics. Research conducted at primary care and KM clinics is valuable as it reflects the reality of community care (Kim and Kang, 2020). However, conducting clinical trials in primary care clinics, especially in KM clinics, has many limitations. Small primary care clinics and KM clinics are chronically short of manpower for crucial manual treatment. Primary care clinics lack equipment and policies regarding the authority of KM doctors and have limited access to modern diagnostic devices. Regarding feasibility to conduct, it requires an adequate research design and outcomes for primary care or KM clinics (Lindbloom et al., 2004). Simple but accurate outcomes with less disturbance to clinics were recommended for this COS.

### 4.3 Outcomes Included in the Core Outcome Set

Effect modifiers were recommended for history taking. Information regarding related diseases, alcohol ingestion, smoking, and concomitant drug use were included as basic history. Moreover, the history of stroke sequelae comprised stroke onset, stroke type, family history of stroke, and rehabilitation treatment. Additionally, since this study targeted elderly individuals with chronic diseases, we decided to record weight, height, and nutritional status, which substantially affect the health of the elderly.

According to a prospective observational study on drug-induced liver damage in patients taking HM, the risk of liver damage due to HM is lower than that with general drugs (Cho et al., 2017). For accurate drug safety evaluation, liver and renal functional markers were included in the COS. Blood aspartate transaminase, alanine transaminase, blood urea nitrogen, and creatinine levels were included. Gamma-glutamyl transferase was proposed to distinguish HM-induced liver damage but was excluded due to its difficulty to test in some KM clinics. Lipid factors, blood sugar, prothrombin time-international normalized ratio, and electrolytes were excluded due to difficulty to test in KM clinics and less relevance with the safety or pathological changes by HM. Since the COS is a minimal set of outcomes, KM doctors may include these outcomes at their discretion if available.

In the early stages after stroke, neurological impairment is dominant. Over time and with neurological recovery, patients tend to show more complicated and overall health problems with decreased neurological symptoms. Therefore, there is a need for a suitable tool that is tailored to the stroke phase. Most stroke-specific measurements, which mainly evaluate neurological impairment, have high reliability and sensitivity for acute or subacute patients but low sensitivity for patients in whom ≥6 months have elapsed since onset (Harrison et al., 2013). The Barthel index, which is a representative scale, is best suited for early stroke survivors requiring intensive rehabilitation. However, it has uncertain reliability in the elderly (Weimar et al., 2002), including those with chronic sequelae. Additionally, the modified Rankin scale (mRS) has limited reliability and substantial interobserver variability. Moreover, there may be a further significant decrease in the reliability of mRS when used by inexperienced primary care doctors (Sainsbury et al., 2005; Harrison et al., 2013). With long-term survival, assessing multifaceted function and the health-related quality of life (HRQoL) becomes important in the chronic stage (Harrison et al., 2013). The 36-item Short Form health-survey questionnaire and Stroke Specific Quality of Life Scale were considered for HRQoL assessment; however, they were excluded given the large numbers of questions that they contain. The EQ-5D-5L and EQ-VAS were selected since they are short and simple tools that have been validated in patients with stroke (Dorman et al., 1997).

It is difficult to specify the scope of stroke sequelae symptoms in the elderly given their diversity across individuals, including motor impairment, cognitive impairment, bladder and bowel dysfunction, infection, pressure sores, deep vein thrombosis, multiple pains, depression, and fatigue (Lui and Nguyen, 2018). MYMOP2, a patient-reported outcome measure that allows patients to select significant symptoms to report, is recommended to assess various symptoms in individuals. This brief and convenient questionnaire is a practical and sensitive measure for patients of complementary medicine and primary care (Paterson, 1996; Ralph and Webley, 2019). Experts selected a symptom evaluation method based on MYMOP2. They offered a list of major symptoms of stroke sequelae to help elderly patients select their symptoms and made each patient select two symptoms that they would like to improve with HM treatment. General health status was included in the symptom list, which made it possible to evaluate general symptoms caused by chronic illness (dyspepsia, sleep disturbance, etc.) and neurological symptoms directly caused by stroke (hemiplegia, dizziness, etc.). This list was composed by experts, and it covers symptoms commonly found in clinical practice. Patients can also select and respond to the symptoms they want to measure, allowing it to serve as a bottom-up survey. A 7-point scale was modified to a numeric rating scale to raise sensitivity. Additionally, a treatment satisfaction questionnaire was recommended to assess effectiveness.

### 4.4 Implications for Outcomes

KM-related outcomes, such as pulse or tongue diagnosis, were not included in the COS for assessing the effectiveness of KM herbal treatments. This might have been due to the difficulty in proving the mechanism of KM treatment and the fact that it is possible to indirectly determine the effect of KM treatment symptomatically. Traditional medicine-related outcomes tend to be overlooked in COS development, highlighting the need for improvements in the quality of traditional medicine outcome measures (Zhang et al., 2021). The development of quantitative measuring methods for KM outcomes may be helpful. Future studies should include sufficiently improved KM outcome measures to the COS in order to reflect crucial KM characteristics.

### 4.5 Implications for Development Process

Our study has several limitations. We performed an overview of SR and analyzed the included studies from two eligible SRs in the literature review process of Phase 1. Furthermore, grey literature was not included in this review scope. Therefore, we cannot be certain that all relevant trials were located. The second limitations include the paucity and often suboptimal quality of primary data. Although the included RCTs have a low risk of bias (Supplementary Figure S2), the analyzed SRs have a very low quality (Supplementary Table S2). Another limitation of this COS development process was the insufficiency of panel recruitment. Although panelists were recruited from a professional society, the number of panelists was insufficient when compared to that of other similar studies. While the involvement of all possible stakeholders in COS development is encouraged, our study involved only disease specialists and KM primary care clinicians. To supplement this, primary clinicians were asked to answer questions after applying the COS to patients. A semi-structured interview with patients and policy experts is recommended to refine the COS in further studies.

### 4.6 Conclusion

The COS-SS-KM is an outcome set for evaluating the effectiveness and safety of herbal KM treatment for stroke sequelae in elderly patients at primary clinics. This is the first COS developed for evaluating the effectiveness of herbal KM treatment in primary care and may evaluate the effectiveness of HM, which is difficult to capture with ordinary outcomes. This COS will be applied to evaluate health insurance pilot projects with continuous modifications for evaluating expanded projects. Future studies should supplement the COS by reflecting the opinions of other stakeholders, including patients and policy experts.

## Data Availability

The original contributions presented in the study are included in the article/Supplementary Material, further inquiries can be directed to the corresponding author.

## Abbreviations

COMET, Core Outcome Measures for Effectiveness Trials; COS, core outcome set; COS-SS-KM, Korean medicine core outcome set for stroke sequelae; CVR, ontent validity ratio; EQ-5D-5L, ive-level EuroQol 5-D; EQ-VAS, EuroQol Visual Analogue Scale; HM, herbal medicine; HRQoL, health-related quality of life; KM, Korean medicine; mRS, modified Rankin scale; MYMOP2, Measure Yourself Medical Outcome Profile 2; PMG, project management group; QOL, quality of life; RCT, randomized controlled trial; SR, systematic review.
