# Supplementary material for: Development of the Korean Medicine Core Outcome Set for Stroke Sequelae: Herbal Medicine Treatment of Elderly Patients With Stroke Sequelae in Primary Clinics
Source: Front Pharmacol. 2022 Apr 25;13:868662. doi: 10.3389/fphar.2022.868662 (PMC9081499; doi:10.3389/fphar.2022.868662)
Supplement: Supplementary file 1 [file DataSheet1.docx]

**Supplementary 1**. Summary of literature review

**1. Methods**

**1.1. Searched databases:** MEDLINE (via PubMed), and the Cochrane Central Register of Controlled Trials (CENTRAL), Oriental Medicine Advanced Searching Integrated System [OASIS], and Science-On

**1.2. Searching date:** from January 1, 2016 to November 15, 2020

**1.3. Search strategies**

*1.3.1. Medline (via PubMed)*

- (("medicine, chinese traditional"[MeSH Terms] OR ("medicine"[All Fields] AND "chinese"[All Fields] AND "traditional"[All Fields]) OR "chinese traditional medicine"[All Fields] OR ("traditional"[All Fields] AND "chinese"[All Fields] AND "medicine"[All Fields]) OR "traditional chinese medicine"[All Fields] OR ("herbal medicine"[MeSH Terms] OR ("herbal"[All Fields] AND "medicine"[All Fields]) OR "herbal medicine"[All Fields])) AND ("geriatric"[All Fields] OR "geriatrics"[MeSH Terms] OR "geriatrics"[All Fields]) AND "systematic review"[Filter]) AND ((systematicreview[Filter]) AND (2016:2020[pdat]))
- (("medicine, chinese traditional"[MeSH Terms] OR "herbal medicine"[MeSH Terms]) AND ("geriatric"[All Fields] OR "geriatrics"[MeSH Terms] OR "geriatrics"[All Fields]) AND "systematic review"[Filter]) AND ((systematicreview[Filter]) AND (2016:2020[pdat]))

*1.3.2. Cochrane Central Register of Controlled Trials (CENTRAL)*

- "Traditional Chinese medicine" OR "herbal medicine" AND "geriatrics"

in *Cochrane Database of Systematic Reviews*

- #1. MeSH descriptor: [Medicine, East Asian Traditional] explode all trees

#2. MeSH descriptor: [Geriatrics] explode all trees

# 3. #1 AND #2

#4. Systematic review

#5. #3 AND #4

*1.3.3. OASIS*

- 한약 AND 노인 (Korean)
- 한약 AND 고령 (Korean)

*1.3.4. Science-On*

- 한약 AND 노인 (Korean)
- 한약 AND 고령 (Korean)

**1.4. Inclusion criteria:**

- Participants: patients with stroke sequelae (over 6 months from onset, ages > 65)

- Intervention: herbal medicine (Traditional Korean Medicine or Traditional Chinese Medicine)

- Comparison: conventional drugs

- Outcomes: effectiveness (symptoms, quality of life, physical test), safety (laboratory test, physical test)

- Study design: Systematic review and meta-analysis

**2. Results**

A total of one SR was included in this review. The final selected one SR [1] included 5 RCTs [2-6] (Figure S1 and Table S1). Furthermore, this SR [1] referred to the information about previous published SR [7] which included 4 eligible RCTs [8-11] (Figure S1 and Table S1). Therefore, a total of 9 RCTs was analyzed as listed in Table S1.

**Identification of studies via databases**

Duplicated records removed (n=3)

Records identified (n=56) from:

PubMed (n=29)

CENTRAL (n=15)

OASIS (n=0)

Science-On (n=12)

**Identification**

Records excluded (n=33):

Not SR (n=21)

Not herbal medicine (n=12)

Titles and abstracts screened (n=53)

**Screening**

Records excluded (n=19)

Not include stroke (n=19)

Full-text articles assessed for eligibility (n = 20)

SR included in the analysis (n=1)

(5 RCTs and 1 SR were analysis)

**Included**

9 RCTs included in the analysis

**Figure S1.** Flow chart of selection process. SR: systematic review; RCT: Randomized controlled trial.

**Table S1.** Characteristics of original randomized controlled trials for stroke.

| Authors (year) [ref] | Inclusion criteria | | Target symptom | Outcomes | |
| --- | --- | --- | --- | --- | --- |
|  | Age (years) | Stroke onset |  | Effectiveness | Safety |
| Li (2008) [2]† | Not reported | Within 6 weeks |  | HDS, BI | LFT, RFT, V/S, ECG, AE |
| Chen (2009) [3]† | 18–70 | Between 15 days and 6 months |  | DTER | LFT, RFT, AE |
| Chen (2013) [4]† | ≥ 18 | Within 72 hours |  | mRS, NIHSS, BI, MMSE |  |
| Numata (2014) [5]† | 20–99 | Over 6 months |  | BI, CSS, GVS by abdominal radiography | LFT, RFT, |
| Venketasubramanian (2015) [6]† | ≥ 18 | Within 72 hours |  | mRS, BI |  |
| Kong. (2009) [8]‡ | 21–80 | Within 1 month | Post-stroke symptoms  related to activities of daily living | FIM, FMA, NIHSS |  |
| Harandi, (2011) [9]‡ | 30–72 | Within 1 month |  | FMA |  |
| Shahripour (2011) [10]‡ | 60–80 | Within 1 week |  | BI, mRS, MCA blood flow velocity |  |
| Ghandehari (2011) [11]‡ | ≥ 18 | Not reported |  | Visual field defect |  |
| †Studies were included in Takayama (2017) [1], and ‡Siddiqui (2013) [7].  AE: adverse event; BI: Barthel index; CSS: constipation scoring system; DTER: diagnostic therapeutic effects of apoplexy; ECG: electrocardiogram; FIM: functional independence measure; FMA: Fugl -Meyer assessment scores; GVS: gas volume score; HDS: Hamilton depression scale; LFT: liver function test; MCA: middle cerebral artery; MMSE: mini-mental state examination; mRS: modified Rankin scale; NIHSS: national institutes of health stroke scale; PMG: project management group; RCT: randomized controlled trial; RFT: renal function test; SR: systematic review; V/S: vital signs. | | | | | |


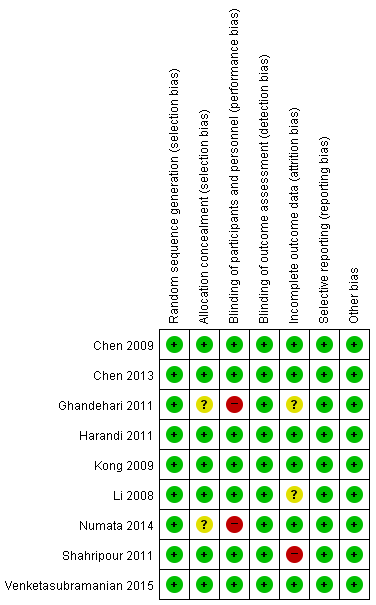

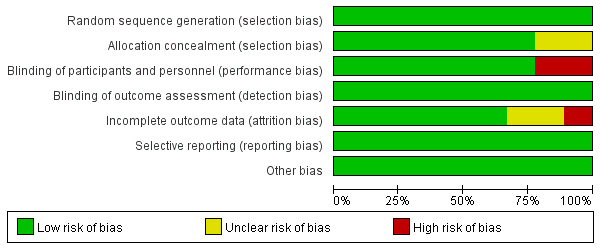


**Figure S2.** Risk of bias: authors’ judgements and percentages about each risk of bias item for included studies

**Table 2S. Assessment of all included systematic reviews using AMSTAR 2.**

| **First author (year)** | **1** | **2** | **3** | **4** | **5** | **6** | **7** | **8** | **9** | **10** | **11** | **12** | **13** | **14** | **15** | **16** | **Rating overall confidence*** |
| --- | --- | --- | --- | --- | --- | --- | --- | --- | --- | --- | --- | --- | --- | --- | --- | --- | --- |
| Takayama (2017) [1] | N | N | N | PY | N | N | N | N | N | Y | No meta-analysis conducted | No meta-analysis conducted | N | N | No meta-analysis conducted | Y | Very Low |
| Siddiqui (2013) [7] | Y | N | Y | PY | Y | N | N | PY | Y | Y | N | N | N | N | N | Y | Very Low |

Y: Yes; PY: Partial Yes; N: No; NMA: No meta-analysis conducted.

Important items 2, 4, 7, 9, 11, Questions 13 and 15 are of very low quality if two or more of these items are omitted, low quality if one important item is omitted, moderate quality if two or more of the non-critical items other than the above important items are omitted, no missing items or non-important If one item is omitted, it is rated as excellent quality.

Domains:

1. Did the research questions and inclusion criteria for the review include the components of PICO?

2. Did the report of the review contain an explicit statement that the review methods were established prior to the conduct of the review and did the report justify any significant deviations from the protocol?

3. Did the review authors explain their selection of the study designs for inclusion in the review?

4. Did the review authors use a comprehensive literature search strategy?

5. Did the review authors perform study selection in duplicate?

6. Did the review authors perform data extraction in duplicate?

7. Did the review authors provide a list of excluded studies and justify the exclusions?

8. Did the review authors describe the included studies in adequate detail?

9. Did the review authors use a satisfactory technique for assessing the risk of bias (RoB) in individual studies that were included in the review?

10. Did the review authors report on the sources of funding for the studies included in the review?

11. If meta-analysis was performed did the review authors use appropriate methods for statistical combination of results?

12. If meta-analysis was performed, did the review authors assess the potential impact of RoB in individual studies on the results of the meta-analysis or other evidence synthesis?

13. Did the review authors account for RoB in individual studies when interpreting/discussing the results of the review?

14. Did the review authors provide a satisfactory explanation for, and discussion of, any heterogeneity observed in the results of the review?

15. If they performed quantitative synthesis did the review authors carry out an adequate investigation of publication bias (small study bias) and discuss its likely impact on the results of the review?

16. Did the review authors report any potential sources of conflict of interest, including any funding they received for conducting the review?

**Reference**

[1] Takayama, S., and Iwasaki, K. (2017). Systematic review of traditional Chinese medicine for geriatrics. Geriatr. Gerontol. Int. 17, 679–688.

[2] Li, L. T., Wang, S. H., Ge, H. Y., et al. (2008). The beneficial effects of the herbal medicine Free and Easy Wanderer Plus (FEWP) and fluoxetine on post-stroke depression. J. Altern. Complement. Med. 14, 841–846.

[3] Chen, C., Venketasubramanian, N., Gan, R. N., et al. (2009). Danqi Piantang Jiaonang (DJ), a traditional Chinese medicine, in poststroke recovery. Stroke, 40, 859–863.

[4] Chen, C. L., Young, S. H., Gan, H. H., et al. (2013). Chinese medicine neuroaid efficacy on stroke recovery: a double-blind, placebo-controlled, randomized study. Stroke, 44, 2093–2100.

[5] Numata, T., Takayama, S., Tobita, M., et al. (2014). Traditional Japanese medicine daikenchuto improves functional constipation in poststroke patients. Evid. Based. Complement. Alternat. Med. 2014.

[6] Venketasubramanian, N., Young, S. H., San Tay, S., et al. (2015). Chinese medicine NeuroAiD efficacy on stroke recovery-extension study (CHIMES-E): a multicenter study of long-term efficacy. Cerebrovasc. Dis. 39, 309–318.

[7] Siddiqui, F. J., Venketasubramanian, N., Chan, E. et al. (2013). Efficacy and safety of MLC601 (NeuroAiD®), a traditional Chinese medicine, in poststroke recovery: a systematic review. Cerebrovasc. Dis. 35(Suppl. 1), 8–17.

[8] Kong, K. H., Wee, S. K., Ng, C. Y., et al. (2009). A double-blind, placebo-controlled, randomized phase II pilot study to investigate the potential efficacy of the traditional Chinese medicine Neuroaid (MLC 601) in enhancing recovery after stroke (TIERS). Cerebrovasc. Dis. 28, 514–521.

[9] Harandi, A. A., Abolfazli, R., Hatemian, A., et al. (2011). Safety and efficacy of MLC601 in Iranian patients after stroke: a double-blind, placebo-controlled clinical trial. Stroke. Res. Treat. 2011.

[10] Shahripour, R. B., Shamsaei, G., Pakdaman, H., et al. (2011). The effect of NeuroAiD™(MLC601) on cerebral blood flow velocity in subjects' post brain infarct in the middle cerebral artery territory. Eur. J. Intern. Med. 22, 509–513.

[11] Ghandehari, K., Mood, Z. I., Ebrahimzadeh, S., et al. (2011). NeuroAid (MLC601) versus piracetam in the recovery of post-infarct homonymous hemianopsia. Neural Regen Res, 6, 418–422.
